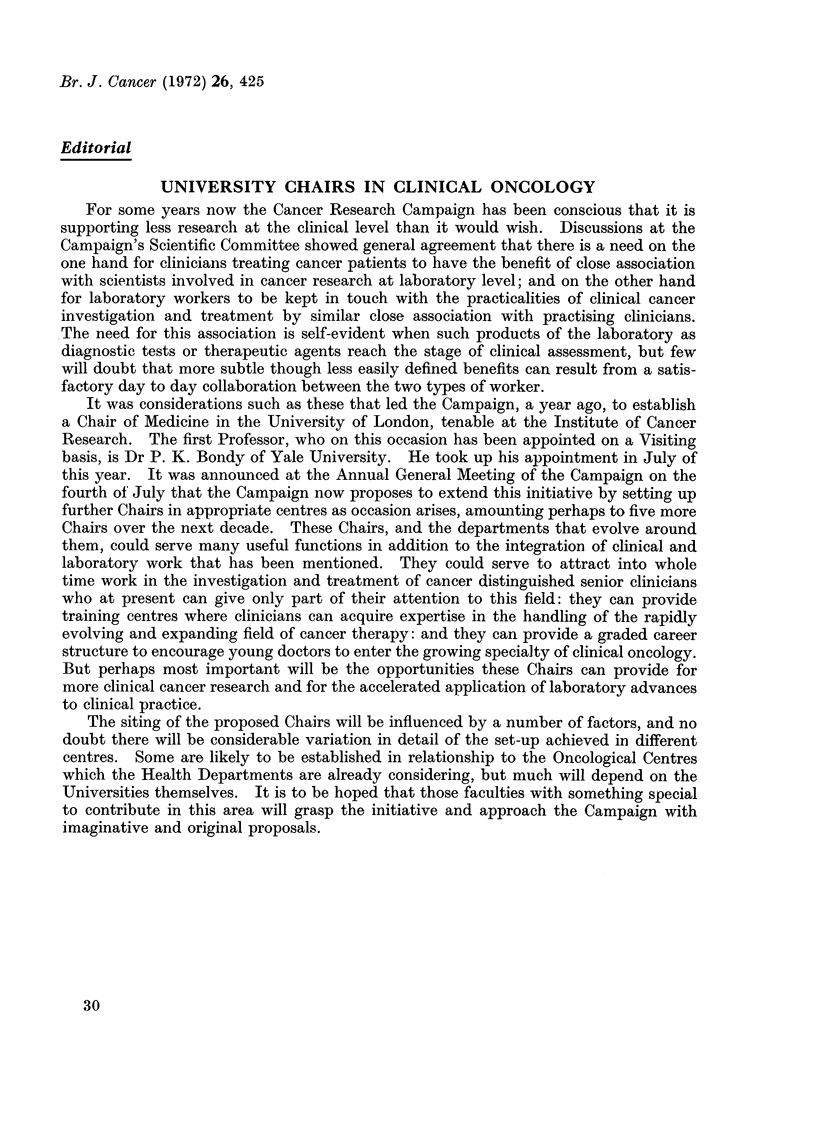# University Chairs in Clinical Oncology

**Published:** 1972-12

**Authors:** 


Br. J. Cancer (1972) 26, 425

*Editorial*

### UNIVERSITY CHAIRS IN CLINICAL ONCOLOGY

For some years now the Cancer Research Campaign has been conscious that it is supporting less research at the clinical level than it would wish. Discussions at the Campaign's Scientific Committee showed general agreement that there is a need on the one hand for clinicians treating cancer patients to have the benefit of close association with scientists involved in cancer research at laboratory level; and on the other hand for laboratory workers to be kept in touch with the practicalities of clinical cancer investigation and treatment by similar close association with practising clinicians. The need for this association is self-evident when such products of the laboratory as diagnostic tests or therapeutic agents reach the stage of clinical assessment, but few will doubt that more subtle though less easily defined benefits can result from a satisfactory day to day collaboration between the two types of worker.

It was considerations such as these that led the Campaign, a year ago, to establish a Chair of Medicine in the University of London, tenable at the Institute of Cancer Research. The first Professor, who on this occasion has been appointed on a Visiting basis, is Dr P. K. Bondy of Yale University. He took up his appointment in July of this year. It was announced at the Annual General Meeting of the Campaign on the fourth of July that the Campaign now proposes to extend this initiative by setting up further Chairs in appropriate centres as occasion arises, amounting perhaps to five more Chairs over the next decade. These Chairs, and the departments that evolve around them, could serve many useful functions in addition to the integration of clinical and laboratory work that has been mentioned. They could serve to attract into whole time work in the investigation and treatment of cancer distinguished senior clinicians who at present can give only part of their attention to this field: they can provide training centres where clinicians can acquire expertise in the handling of the rapidly evolving and expanding field of cancer therapy: and they can provide a graded career structure to encourage young doctors to enter the growing specialty of clinical oncology. But perhaps most important will be the opportunities these Chairs can provide for more clinical cancer research and for the accelerated application of laboratory advances to clinical practice.

The siting of the proposed Chairs will be influenced by a number of factors, and no doubt there will be considerable variation in detail of the set-up achieved in different centres. Some are likely to be established in relationship to the Oncological Centres which the Health Departments are already considering, but much will depend on the Universities themselves. It is to be hoped that those faculties with something special to contribute in this area will grasp the initiative and approach the Campaign with imaginative and original proposals.